# Early pathogenesis during infectious bursal disease in susceptible chickens is associated with changes in B cell genomic methylation and loss of genome integrity

**DOI:** 10.1016/j.dci.2017.03.014

**Published:** 2017-08

**Authors:** Nick A. Ciccone, Lorraine P. Smith, William Mwangi, Amy Boyd, Andrew J. Broadbent, Adrian L. Smith, Venugopal Nair

**Affiliations:** a The Pirbright Institute, Woking, GU24 0NF Surrey, United Kingdom; b Department of Zoology, University of Oxford, OX1 3PS Oxfordshire, United Kingdom

**Keywords:** B-cell, T-cell, CD40L, Infectious bursal disease virus (IBDV), 5-Hydroxymethylcytosine (5hmC), IBDV, Infectious Bursal Disease Virus, 5hmC, 5-hydroxymethylcytosine, 5mC, 5-methylcytosine, Tet, Ten-11 translocation, BF, bursa of Fabricius, RIR, Rhode Island Red, 15l, line 15I

## Abstract

We propose a model by which an increase in the genomic modification, 5-hydroxymethylcytosine (5hmC), contributes to B cell death within the chicken bursa of Fabricus (BF) infected with infectious bursal disease virus (IBDV). Our findings indicate that, following an IBDV infection, Rhode Island Red (RIR) chickens have fewer surviving B cells and higher levels of 5hmC in the BF than the more resistant 15l line of birds. Elevated genomic 5hmC levels within the RIR BF are associated with markers of immune responses: infiltrating T cells and increased expression of CD40L, FasL and iNOS. Such changes correlate with genomic fragmentation and the presence of IBDV capsid protein, VP2. To explore the effects of CD40L, the immature B cell line, DT40, was exposed to recombinant chicken CD40L that resulted in changes in nuclear 5hmC distribution. Collectively, our observations suggest that T cell infiltration exacerbates early immunopathology within the BF during an IBDV infection contributing to B cell genomic instability and death to facilitate viral egress and immunosuppression.

## Highlights

• Genomic 5-hydroxymethylation (5hmC) is studied in the context of susceptible and resistant IBDV chickens.
• Increased T-cell infiltration is associated with 5hmC in infected tissue.
• 5hmC correlates with cell death and the presence of IBDV protein, VP2.
• T-cell CD40L could contribute to B-cell death through a 5hmC dependent mechanism.

## Graphical abstract

**Figure:**
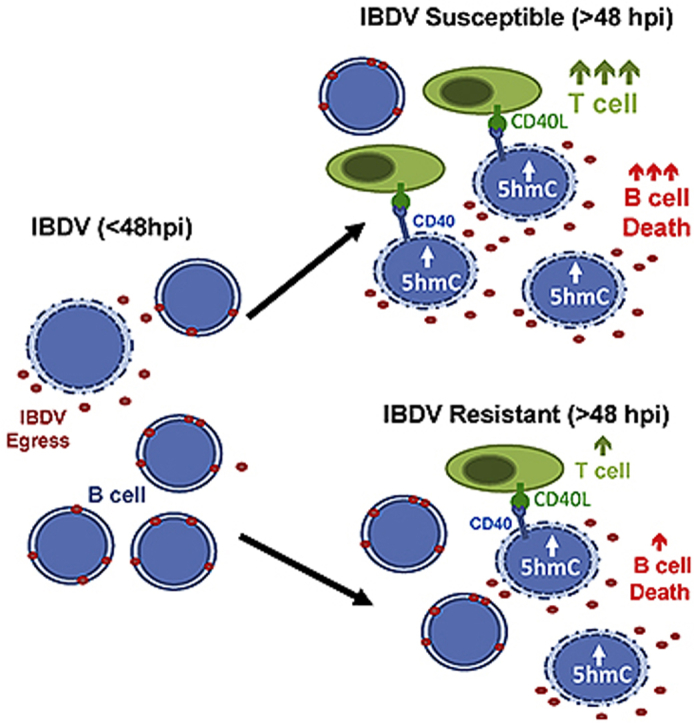

## 1 Introduction

The gut associated lymphoid tissue (GALT) is a critical site for B cell development for several species, including the illeal Peyer's patches (IPP) of cattle and sheep, the appendix of the rabbit, and most famously the bursa of Fabricius (BF) in birds (Parra et al., 2013, Ratcliffe, 2006). Infectious bursal disease virus (IBDV) causes significant immunosuppression in commercial chicken flocks through elimination of a substantial pool of maturing B cells (Ingrao et al., 2013, Sharma et al., 2000), leaving infected birds susceptible to a wide range of other pathogenic infections and poorly responsive to vaccination (Ingrao et al., 2013, Sharma et al., 2000). Among the mysteries of IBDV pathogenesis is how this non-enveloped virus is released from and kills bursal B cells.

We have proposed that the levels of methylation such as 5-methylcytosine (5mC) and 5-hydroxymethylcytosine (5hmC) are key to protecting the genome in these cells (Ciccone et al., 2014). The modification 5mC is reported to maintain genomic stability and integrity (Hackett and Surani, 2013), and we find that B cells in the BF have high levels of genomic 5mC. A modification involved in complete genomic demethylation, 5-hydroxymethylcytosine (5hmC), along with mRNAs encoding the enzymes that catalyse the formation of 5hmC, Tet1 (Ten-11 translocation 1) and Tet2 (Ciccone et al., 2014) are progressively reduced during bursal B cell development.

We have previously shown that a very virulent strain of IBDV, UK661, disrupts this genomic methylation pattern in the BF of post-hatch chickens by causing a global increase in 5hmC levels as well as Tet1 and Tet2 mRNAs (Ciccone et al., 2014). While some IBDV virions may be released by non-lytic mechanisms (Mendez et al., 2015), the liberation of viable viral particles is thought to be predominantly dependent on an as yet uncharacterised series of events that culminate in cell lysis (Ingrao et al., 2013, Sharma et al., 2000). Given that IBDV infection leads to a disruption of bursal genomic methylation, it is possible that B cell genomic instability and cell death facilitate IBDV egress.

Several investigators have observed infiltration of T cells within the chicken BF during the acute phase of IBDV infection (Kim et al., 2000, Poonia and Charan, 2004, Rauf et al., 2012, Tanimura and Sharma, 1997). The magnitude of this T cell response is greater in birds infected with more virulent IBDV strains, associated with more extensive bursal lesions (Poonia and Charan, 2004), while *in vivo* T cell depletion leads to increased IBDV load within the BF and reduced lesions (Kim et al., 2000). Taken together these observations suggest tissue damage observed within this lymphoid organ is associated with viral virulence and, at least in part, with the T cell response.

In this study, we make use of two well characterised, genetically distinct lines of chicken that are either resistant (line 15I) or susceptible (Rhode Island Red, RIR) to mortality associated with IBDV infection (Bumstead et al., 1993), in order to test the hypotheses that a more favourable outcome of IBDV infection is correlated with reduced BF T cell infiltration, greater numbers of surviving B cells and lower levels of the genomic modification 5-hydroxymethylcytosine (5hmC). We also identify increased levels of CD40 ligand (CD40L) within the BF of IBDV susceptible birds and explore if this molecule can alter B cell genomic 5hmC levels. The results allow us to propose a model by which CD40L-expressing cells within IBDV-infected bursa can lead to modifications to genomic methylation that exacerbates bursal B cell death and release of IBDV virions.

## 2 Material and methods

### 2.1 Animals and IBDV infection

Specific pathogen free (SPF), Line 15I and Rhode Island Red (RIR) chickens were obtained from the poultry production unit at The Pirbright Institute. Both rearing and experimentation were conducted in accordance with the UK Animal Scientific Procedures act 1986, approved by the Pirbright Institute internal ethical review procedure, and performed under Home Office guidelines of the United Kingdom. IBDV infections were performed as described previously (Ciccone et al., 2014) using a very virulent strain, UK661. Five birds were culled at various time points: 6, 12, 24 and 48 h post infection (hpi) and the BF were rapidly dissected, cut into equal portions and either frozen directly on dry ice, for protein and DNA studies, or submerged in Optimum Cutting Temperature (OCT) embedding medium, for bioimaging analysis, before freezing and storage at −80^C^ prior to processing.

### 2.2 Cell culture

DT40 cells (a kind gift from Dr. Julian Sale, MRC Laboratory for Molecular Biology, Cambridge) were cultured in RPMI 1640 with l-glutamine (Gibco) supplemented with 10% fetal calf serum (Sigma), 1% chicken serum (Sigma) and 50 μM β-mercaptoethanol (Gibco). HD11 cells (Beug et al., 1979) were maintained in RPMI 1640 with l-glutamine (Gibco) supplemented with 8% fetal calf serum (Sigma) and 2% chicken serum (Sigma). Both cell lines were incubated at 38.5 °C with 5% CO_2_. CD40L, used to stimulate cultured cells, was produced and purified as described elsewhere (Kothlow et al., 2008, Tregaskes et al., 2005). DT40 cell were stimulated with 500 ng/mL recombinant chicken CD40L or vehicle for 24 h.

### 2.3 Immunofluorescence, bioimaging and analysis

Detailed methodologies for immunofluorescence and bioimaging of BF tissue are described elsewhere (Ciccone et al., 2014). A mouse anti-chicken CD3 antibody (Southern Biotech) was diluted 1:250 in PBS with 10% FCS to determine T-cells within the BF. Alexa Fluro 568 donkey anti-mouse secondary antibody (Life Technologies) was diluted 1:500. For studies with DT40 cells, methods were followed as outlined for sectioned BF tissue but with the additional step of adhering these suspension cells onto coverslips using Cell-TAK (Corning) following manufacturer instructions. DT40 images were taken from a single focal plane and at roughly the same points within each cell. For quantification of immunoflorescent CD3^+^ cells within individual bursal follicles the original confocal images were converted to 16-bit black and white formats and analysed by imageJ using the free draw tool to delineate individual bursal follicles. After background corrections, integrated density values were normalised by corresponding follicle area and values expressed as arbitrary units.

### 2.4 Genomic DNA analysis

The protocol for DNA dot blot assays to determine genomic 5hmC levels was performed as described previously (Ciccone et al., 2014) with the following modifications: genomic DNA from approximately 20 mg of frozen BF tissue was purified using a tissue and blood genomic DNA purification kit (Qiagen). A known concentration of genomic DNA was arrayed on a nitrocellulose membrane. For studies analysing genomic DNA integrity, 1 μg of purified DNA was electrophoresed in a 2% agarose gel run in 1x Tris/Borate/EDTA (TBE) buffer (Life Technologies) and stained with ethidium bromide before analysis using a GelDoc EZ imager (BioRad).

### 2.5 Quantitative reverse transcriptase real-time PCR (qRT-PCR) assay

For BF samples, total RNA extraction and subsequent qRT-PCR was performed as outlined elsewhere (Ciccone et al., 2014). Glyceraldehyde-3-phosphate dehydrogenase (GAPDH) was used as a reference gene and the ΔΔCt method of quantification was performed to obtain relative fold changes in mock infected six-week old BF. Primer sequences for GAPDH, Tet1 and Tet2 were published previously and validated to work efficiently in real time qPCR assays. Primer sequences for the detection of chicken CD40L (forward: CGGGAGAAGATCAGAAGTCG and reverse: TGCCACAGATGTCTCATTCC), FasL (forward: GGGCTGAGCATGTTTCAGAT and reverse: GAGACAGGTTCCCACTCCAA) and iNOS (forward: AGAAGCACTAGAGATGGGCC and reverse: ACTGTTTCTAGTCGGGCCAG) were designed using Primer 3.

### 2.6 Western blot

Western blotting for IBDV VP2 capsid protein was described in detail previously (Ciccone et al., 2014).

### 2.7 Statistical analysis

Student unpaired *t*-test was used to determine statistical significance between mock and infected or stimulated samples using Prism 5 (Graphpad Software).

## 3 Results and discussion

### 3.1 Elevated immune markers within IBDV infected BF of susceptible birds is associated with increased 5hmC levels, genomic fragmentation and viral VP2 protein expression

**Fig. 1:**
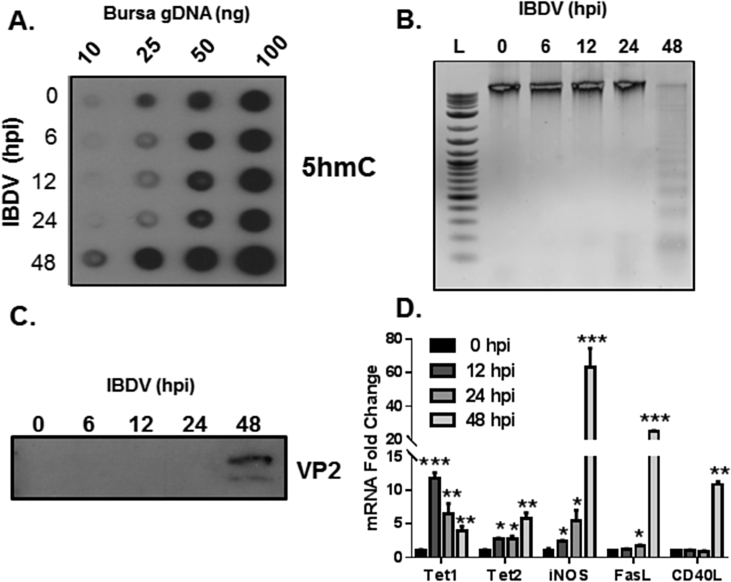
**An increased genomic 5hmC level in IBDV infected bursa is associated with genomic fragmentation and increased expression of Tet1, Tet2 and immune responsive genes**. Dot blot of bursal genomic DNA probed with a specific 5hmC antibody from either mock or IBDV UK661 infected RIR BF taken at various hours post infection (hpi) (A). Visualisation of genomic DNA integrity from mock or IBDV infected RIR BF by ethidium bromide. L = DNA size marker (B). Detection of IBDV VP2 capsid by western blot using protein lysates of RIR BF infected with IBDV UK661 at various time points (C). Fold changes of Tet1, Tet2, iNOS, CD40L and FasL gene expression within the RIR BF infected at various time points with IBDV as measured by real time RT-PCR (D). * = p < 0.05, ** = p < 0.01, *** = p < 0.001 as measured by Student unpaired *t*-test between mock and infected samples.

BF samples from IBDV susceptible RIR birds, taken at different time points after UK661 infection, were analysed to determine the relationship between genomic 5hmC levels and genome integrity. As measured by DNA dot blot, genomic 5hmC levels within the BF of RIR chickens increased substantially after 48 h post infection (hpi) compared to both 5hmC levels within mock infected (0) and birds infected for periods shorter than 48 h (Fig. 1A). Genomic samples used in this dot blot were also analysed by agarose gel electrophoresis which revealed genomic fragmentation, as demonstrated by DNA laddering, at 48 hpi (Fig. 1B). The IBDV viral capsid protein, VP2, an indicator of late stage viral replication, was detected only at 48 hpi as measured by western blot (Fig. 1C). Collectively these findings establish a relationship between elevated genomic 5hmC levels and a loss of genome integrity within the BF following IBDV infection at a stage when mature virions have been produced in preparation for viral egress.

To further investigate the underlying mechanism that causes 5hmC associated B cell death, BF Tet1 and Tet2 transcripts were measured at different time points after UK661 infections. Tet1 transcripts were significantly more abundant within IBDV infected BF at all time points analysed compared to mock infected birds (Fig. 1D). Maximal Tet1 gene expression was detected at 12 hpi with a progressive reduction at 24 and 48 hpi that may be related to a loss of bursal B-cells. There is a modest but significant increase in Tet2 mRNA within the BF of infected birds at 12 and 24 hpi with maximal levels detected at 48 hpi that may be related to immune cells such as macrophages and T cells that are known to infiltrate the BF following an IBDV infection (Rasoli et al., 2015). Collectively this data suggests that Tet1 and 2 are viral responsive genes and that the increased expression of these enzymes mediates the rise in genomic 5hmC levels within the IBDV infected BF.

To explore the notion that observed genomic changes within the BF could be mediated by infiltrating macrophages and/or T cells we determined BF gene expression of immune markers that are indicators of either macrophage (iNOS) or T cell (FasL and CD40L) activation. iNOS mRNA levels gradually increased during IBDV infection also reaching maximal levels at 48 hpi (Fig. 1D). iNOS dependent production of reactive nitrogen species (RNS), such as nitric oxide (NO), can cause genomic instability through epigenetic mechanisms in a number of biological systems (Mikhed et al., 2015). Further, a characteristic feature of bursal B cells is their ability to rapidly divide; this feature may make these lymphocytes more susceptible to cytotoxic NO effects as previous observations have shown that pathogen induced NO is more effective at killing fast growing cells (Mocca and Wang, 2012). Collectively, this supports the idea that iNOS could play an important role during an IBDV infection and it is conceivable that NO can increase 5hmC levels to facilitate BF genomic instability.

It is also conceivable that an increase in BF 5hmC levels is mediated by T cell dependent signaling. Activated T cells may interact with other immune cells including B cells through a range of secreted and cell surface signaling factors, and while cytokine responses have been analysed in IBDV infections (Liu et al., 2010, Wang et al., 2014), the levels of cell surface molecules have been given less attention. We found that both FasL and CD40L mRNA levels were significantly increased within 48 hpi in BF samples from RIR birds compared to mock infected RIR birds, with no significant increase in either mRNA between mock and infected BF in samples taken prior to 48 hpi (Fig. 1D). Our finding is consistent with the report that FasL, a member of the tumour necrosis factor (TNF) super family, is increased at both the mRNA and protein level within the BF and spleen of IBDV infected chickens (Rauf et al., 2012). These investigators proposed that infiltration of CD8^+^ T cells, bearing FasL, engage with cognate Fas receptor on IBDV infected B cells to induce cytolytic effects that ultimately clear the viral infection (Rauf et al., 2012). The kinetics of FasL mRNA upregulation (Fig. 1D) coincides with the death of B cells as indicated by genomic laddering (Fig. 1B), further supporting a role for the Fas-FasL pathway in the clearance of IBDV infected cells.

Extending this idea, we discovered that CD40L, another member of the TNF superfamily, is significantly increased after 48 hpi (Fig. 1D), suggesting that this T cell ligand could have cytolytic action on IBDV infected B cells. In support of this view, it has been observed that a recombinant chicken CD40L can cause cell death in an immature chicken B cell model, the DT40 cell line (Tregaskes et al., 2005).

### 3.2 Effects of IBDV infection on B cell numbers, T cell infiltration and 5hmC levels within the BF of susceptible and resistant chickens

**Fig. 2:**
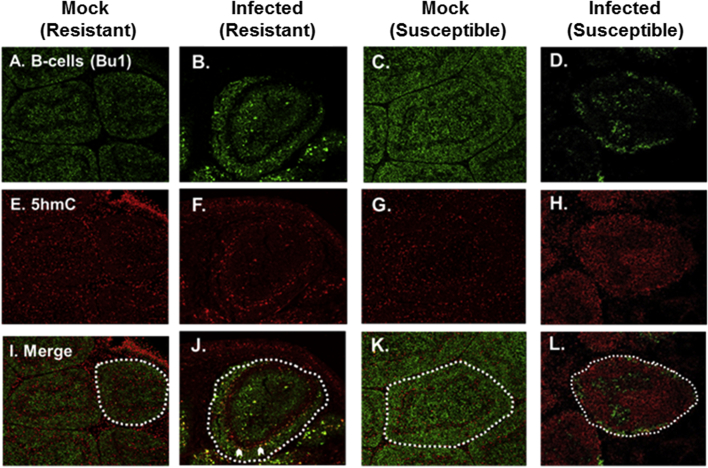
**Loss of Bu-1+ B-cells within the BF of IBDV susceptible RIR birds after IBDV infection (48hpi) is associated with increased genomic 5hmC levels**. Comparative levels of the B cell marker, Bu-1 (green) and 5hmC (red) within sectioned BF of mock (A, E, I) and infected (B, F, J) resistant 15l or mock (C, G, K) and infected (D, H, L) susceptible RIR as measured by immunofloresence. Individual bursal follicles are delineated within white dashed lines. White chevrons indicate yellow staining that suggests B cells expressing both Bu-1 and increased 5hmC levels.

Sectioned BF from either IBDV UK661 or mock infected six-week old 15l (resistant) or RIR (susceptible) birds were co-stained with specific antibodies for Bu-1, an avian B cell marker, and the genomic modification 5hmC. Immunofluorescence analysis indicates that, after 48 hpi, Bu-1 signal was considerably higher in 15l (Fig. 2B) than in RIR (Fig. 2D) bursal follicles. The more extensive depletion of Bu-1+ B-cells within infected RIR was associated with a greater increase in 5hmC levels (Fig. 2H) when compared to levels measured within both mock infected RIR (Fig. 2E) and 15l (Fig. 2G) respectively, and also infected 15l bursal follicles (Fig. 2F). Cells with brighter 5hmC may be B cells that have lost Bu-1 expression; in support of this view, reticuloendotheliosis virus T downregulates Bu-1 in infected B cells (V. Nair, unpublished observation), and this may also be the case for IBDV infection. Alternatively, increased 5hmC intensity may pertain to another immune cell type such as infiltrating and/or activated T cells, macrophages and dendritic cells.

**Fig. 3:**
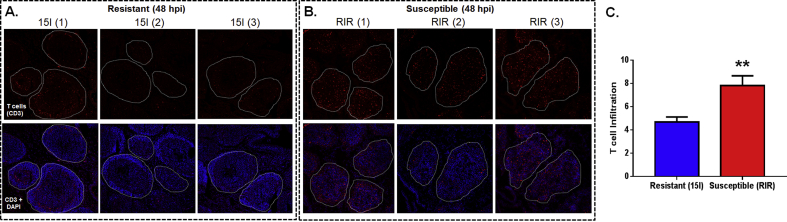
**IBDV infection (48 hpi) causes greater T cell infiltration within the bursa of IBDV susceptible (RIR) than resistant (15l) infected birds**. Sectioned BF of resistant 15l (A) and susceptible RIR (B) stained with the pan T cell marker, CD3 (red), and DAPI (blue) used for counterstaining double stranded DNA. Individual bursal follicles are delineated within white dashed lines. Comparison of T cell infiltration within 15l and RIR BF, as measured by fluorescent CD3^+^ cells, is represented as a bar graph. **p < 0.01 as determined by student unpaired *t*-test.

Staining with anti-CD3, which recognises all avian T cell subsets, indicates the presence of these lymphocytes within the BF of both 15l and RIR chickens after 48 hpi of IBDV (Fig. 3A and B). This finding complements previous studies (Kim et al., 2000, Poonia and Charan, 2004, Rauf et al., 2012, Tanimura and Sharma, 1997) and shows significantly more T cells within bursal follicles of infected RIR than 15l birds (Fig. 3C). With the associated increase in 5hmC levels and a greater comparative loss of Bu-1+ B cells, this observation suggests that T cells may contribute to the IBDV susceptible RIR phenotype.

It remains to be established if T cell infiltration within the IBDV infected BF is a consequence or cause of B cell death. It is possible that the initial cause is predominantly T cell independent, with bursal infiltration of these lymphocytes occurring due to the presence of cellular detritus acting as danger associated molecular patterns (DAMPs) or alarmins and cytokine/chemokine release as a consequence of inflammation and cell death (Rock et al., 2011). In this scenario, an autonomous cellular mechanism, such as IBDV induced B cell necroptosis, could act as a signal for T cell infiltration. Given our current observations, together with previous studies of other researchers, it is equally plausible that infiltrating T cells actively participate in events that culminate in B cell lysis and release of IBDV virions. T cell infiltration into the BF has been observed as rapidly as one-day post IBDV infection (Tanimura and Sharma, 1997) and so are appropriately located to influence acute stages of IBDV pathogenesis.

In mice, activation of cytotoxic T cells is augmented by the presence of intracellular components released upon cell death that act as adjuvants to accelerate immune responses (Matzinger, 2002, Shi et al., 2000). This could be relevant in early pathological changes during an IBDV infection where antigen processing and presentation is required to stimulate cytotoxic αβT cells. Once activated αβT cells could exacerbate BF lesions and facilitate IBDV egress and cause extensive immunopathology. Although this is a plausible mechanism, it remains to be shown that 48 hpi of IBDV is sufficient to generate a classic αβT cell immune response that involves epitope presentation. Regardless, after 48 hpi, the increase in CD40L and FasL mRNAs within the BF (Fig. 1D) suggests that infiltrating T cells are activated but it remains unclear which T cell subset could be expressing these ligands. As γδT cells make up a significant portion of the total T cell population within chickens (Bucy et al., 1991), It is possible this T cell subtype is activated and mediating cytolytic effects through either CD40L or FasL. This is a plausible mechanism as both ligands have been shown to be functionally expressed on mammalian γδT cells (Horner et al., 1995, Huber, 2000, Roessner et al., 2003). The innate-like abilities of γδT cells (Born et al., 2007) further supports this notion as these lymphocytes would not require antigen presentation for activation allowing for a more rapid immune response. In fact, γδT cell levels significantly increase in the gut of chickens infected with *Salmonella* Typhimurium at 48hpi (Pieper et al., 2011). Furthermore, the presence of these immune cells have been observed within IBDV infected BF (Tanimura and Sharma, 1997), collectively these observations suggest a possible role for γδT cells during early pathogenesis of IBDV infections.

### 3.3 Recombinant chicken CD40L alters 5hmC content within the immature chicken B-cell line, DT40

To investigate if CD40L could alter 5hmC levels in chicken B cells we made use of trimerised, recombinant chicken CD40L (Kothlow et al., 2008, Tregaskes et al., 2005) and the B cell line, DT40, which was derived from the BF of an avian leukosis virus (ALV) infected chicken (Baba et al., 1985). This approach circumvents the complication of studying *in vitro* bursal B cells that require supplemental support, such as CD40L, to survive in culture thus making the study of how IBDV infected B cells respond to CD40L problematic. A substitute molecule that can potentially support B cell survival is B cell activating factor of the TNF family (BAFF) (Kothlow et al., 2007, Schneider et al., 2004), but this approach is fraught with the unknown effects BAFF has on cultured primary B cells. An alternative is the DT40 cell line that is inherently infected with a B cell targeting virus, does not require CD40L to survive and has been extensively studied as a B cell model (Baba et al., 1985).

**Fig. 4:**
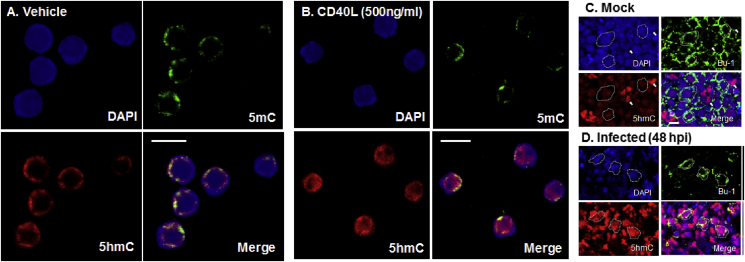
**Recombinant chicken CD40L changes nuclear 5hmC location in the immature B-cell line, DT40.** DT40 cells were stimulated in suspension with 500 ng/mL of recombinant chicken CD40L (A) or vehicle (B) for 24 h. For comparison, bursal sections of RIR either mock infected (C) or infected (D) with IBDV, UK661 (48hpi) are also shown. Dashed lines delineate examples of Bu-1+ B cells while arrows indicate Bu-1- non-B cells. Immunofluorescent images depict staining with 5hmC (red), 5mC (green) and DAPI (blue). Bar = 10 μM.

DT40 cells were exposed to 500 ng/mL chicken CD40L or vehicle for 24 h and subsequently fixed for immunofluorescence studies. Cells were stained with antibodies specific to both 5mC and 5hmC and counterstaining with DAPI revealed nuclei. Vehicle treated DT40 cells have a characteristic 5hmC and 5mC distribution that encircles the periphery of the nucleus in a halo-like pattern (Fig. 4A). After CD40L activation, the distribution of 5hmC within DT40 cells was altered, becoming more widely spread throughout the nucleus (Fig. 4B) while 5mC distribution remained at the periphery of the nucleus although the halo-like distribution was less prominent (Fig. 4B). This redistribution of 5hmC could be indicative of this genomic modification's role in modulating actively transcribed genes. In mammalian cells, parts of the genome that are gene-rich aggregate within the nuclear interior, whereas genomic regions that are gene barren are confined to the periphery (Schneider and Grosschedl, 2007). These findings show that CD40L stimulation of an immature chicken B cell model can alter 5hmC location and have parallels with genomic changes within chicken bursal B cells infected with IBDV (Fig. 4C and D).

Confocal imaging of an uninfected RIR BF, stained with Bu-1 (green) and 5hmC (red), highlights B cells by an encircling green florescence that corresponds to cell surface Bu-1 expression (Fig. 4C). Within these Bu-1+ cells the relative immunoreactive 5hmC levels are lower than adjacent bursal cells that do not express Bu-1 (Fig. 4C) and are unlikely to be B cells. After 48 hpi, Bu-1+ cells decrease dramatically but remaining cells that visibly have some Bu-1 cell surface expression have 5hmC immunofluorescence that is much brighter with a more expansive nuclear distribution (Fig. 4D), than Bu-1+ cells from a mock infected BF (Fig. 4C).

## 4 Conclusion

In this current study we propose a model to explain the events in the BF during early pathogenesis of a very virulent IBDV, UK661 infection. Initially, mature IBDV virions are released in an autonomous mechanism mediated by IBDV that causes B cell lysis. Infiltrating T cells subsequently augment and exacerbate this first wave of B cell death. The death of the host cell, in this case the developing B cell infected with IBDV, may be beneficial to the host if it happens early enough so that virions are not yet produced, or beneficial to the virus, if it happens late enough that it leads to release of virions. Specifically, this study highlights that T cells may contribute to enhanced immunopathology within the BF of IBDV susceptible chickens. Nonetheless, more rapid T cell immune responses may be beneficial in accelerated elimination of IBDV infections without release of virus. This would limit adverse immunosuppressive effects through reduced B cell depletion and could be harnessed in a T cell targeted IBDV vaccine. Alternatively, a therapeutic that minimises T cell BF infiltration or activation could reduce adverse effects caused by T cell dependent immunopathology after an IBDV infection. Future investigations will explore the functional significance of T cell responses through manipulation of distinct T cell populations *in vivo* within the context of IBDV infections.
